# Investigation of Photosystem II Functional Size in Higher Plants under Physiological and Stress Conditions Using Radiation Target Analysis and Sucrose Gradient Ultracentrifugation

**DOI:** 10.3390/molecules27175708

**Published:** 2022-09-05

**Authors:** Maria Teresa Giardi, Amina Antonacci, Eleftherios Touloupakis, Autar K. Mattoo

**Affiliations:** 1 Institute of Crystallography, CNR, Via Salaria Km 29.3, 00016 Monterotondo, Italy; 2 Biosensor Srl, Via Olmetti 44, 00060 Formello, Italy; 3 Research Institute on Terrestrial Ecosystems, CNR, Via Madonna del Piano 10, 50019 Sesto Fiorentino, Italy; 4 USDA-ARS, Sustainable Agricultural Systems Laboratory, Beltsville, MD 20705, USA

**Keywords:** photosystem II, core populations in vitro, radiation target analysis, stress conditions

## Abstract

The photosystem II (PSII) reaction centre is the critical supramolecular pigment–protein complex in the chloroplast which catalyses the light-induced transfer of electrons from water to plastoquinone. Structural studies have demonstrated the existence of an oligomeric PSII. We carried out radiation inactivation target analysis (RTA), together with sucrose gradient ultracentrifugation (SGU) of PSII, to study the functional size of PSII in diverse plant species under physiological and stress conditions. Two PSII populations, made of dimeric and monomeric core particles, were revealed in *Pisum sativum*, *Spinacea oleracea*, *Phaseulus vulgaris*, *Medicago sativa*, *Zea mais* and *Triticum durum*. However, this core pattern was not ubiquitous in the higher plants since we found one monomeric core population in *Vicia faba* and a dimeric core in the *Triticum durum* yellow-green strain, respectively. The PSII functional sizes measured in the plant seedlings in vivo, as a decay of the maximum quantum yield of PSII for primary photochemistry, were in the range of 75–101 ± 18 kDa, 2 to 3 times lower than those determined in vitro. Two abiotic stresses, heat and drought, imposed individually on *Pisum sativum*, increased the content of the dimeric core in SGU and the minimum functional size determined by RTA in vivo. These data suggest that PSII can also function as a monomer in vivo, while under heat and drought stress conditions, the dimeric PSII structure is predominant.

## 1. Introduction

Plant chloroplasts harbor the photosystem II (PSII) supramolecular pigment–protein complex which catalyses the light-induced transfer of electrons from water to plastoquinone. It consists of a water-splitting system (oxygen evolving complex, OEC), a light-harvesting chlorophyll protein complex (LHCII) and a reaction centre. The OEC is localized to the lumen and consists of a cluster of four manganese atoms and three hydrophilic 33, 23 and 16–17 kDa extrinsic proteins. The PSII reaction centre constitutes the D1/D2 protein complex, two Cyt b559 subunits and a few low molecular weight polypeptides [1,2,3,4], while the core comprises of a reaction center and the inner antennae (CP47 and CP43) [5,6,7,8,9,10]. The D1/D2 protein complex is structurally organised into five membrane-spanning helices with binding sites for four to six chlorophylls (Chl), two pheophytins, a non-heme iron, one or two carotenoids, and two quinones, Q_A_ and Q_B_ [11,12]. 

The PSII reaction centre D1 protein of oxygenic phototrophs is pivotal for sustaining photosynthesis. It is also a target for herbicides and herbicide-resistant weeds [2,8]. PSII complexes are present both in monomeric and dimeric forms [13,14,15,16]. The crystal structures of both the monomeric and dimeric PSII core complex have been determined at different resolutions in thermophilic cyanobacteria [1,6,17,18,19]. The PSII monomer has been suggested to be an intermediate form of PSII assembly and repair [20]. It has been argued that isolated dimeric PSII complexes could be a product of the aggregation of PSII monomers since its isolation requires the use of a detergent [21,22,23]. Moreover, the crystallographic model of the PSII dimer at 3.0 Å resolution revealed the presence of six detergent molecules located at the interface of the two monomers [1].

The functional PSII in vivo was proposed to be a monomer based on the observation that lipid deprivation caused conversion of PSII from a monomeric to a dimeric form [22]. Further, only monomeric PSII was detected in soluble cyanobacterial and red algal thylakoids using blue-native polyacrylamide gel electrophoresis. It was shown that energy transfer between PSII units, determined by the sigmoidal fluorescence increase as observed in the purified dimeric PSII, was barely detectable in vivo [22].

It was recently established that the native form of the PSII core is dimeric and forms super-complexes with inner monomeric and outer trimeric light-harvesting complexes. Such structures have been visualized by cryogenic electron microscopy (cryo-EM) within thylakoid membranes at a very low concentration of detergent digitonin [11], and in osmotically shocked thylakoid membranes. However, high-resolution cryo-EM studies of PSII super-complexes of higher plants did not reveal the presence of detergent molecules between PSII monomers [13,16]. These findings strongly imply that PSII core dimers are not artificial structures.

Radiation target analysis (RTA) has previously been utilized to determine the minimum PSII molecular size. RTA relies on structural damage caused by γ-irradiation reflected by a decrease in the biochemical activity [24,25]. In this system, biochemical activity decreases exponentially with radiation dose at a rate directly proportional to the mass of the individual molecules possessing this activity. RTA of PSII particles from *Phormidium laminosum* [26] and *Spinacia oleracea* thylakoids discerned a molecular mass of 120 kDa and 250 kDa for the reaction center and the oxygen evolving complex, respectively [27,28]. 

In this work, we used RTA to determine the functional size of PSII in vivo. We show here that, in comparison to the sucrose gradient ultracentrifugation (SGU) of PSII cores in vitro, PSII in vivo functions also as a monomer under normal conditions. Moreover, under heat and drought stress conditions, the dimeric PSII functional structure is predominant.

## 2. Results

### 2.1. PSII Core Oligomeric Forms from Solubilised PSII Particles of Various Higher Plants 

All the experiments were performed using seedlings of *Pisum sativum* (Pea), and to analyse the possibility of different behaviours, some investigations were carried out with other plant species, namely, *Spinacea oleracea* (Spinach)*, Vicia faba* (Broad beans), *Triticum durum* (Wheat), *Medicago sativa* (Alfalfa), *Phaseulus vulgaris* (Beans) and *Zea mais* (Maize). To study the distribution of PSII oligomeric forms, enriched PSII particles were isolated from seedlings of the various plant species.

**Figure 1:**
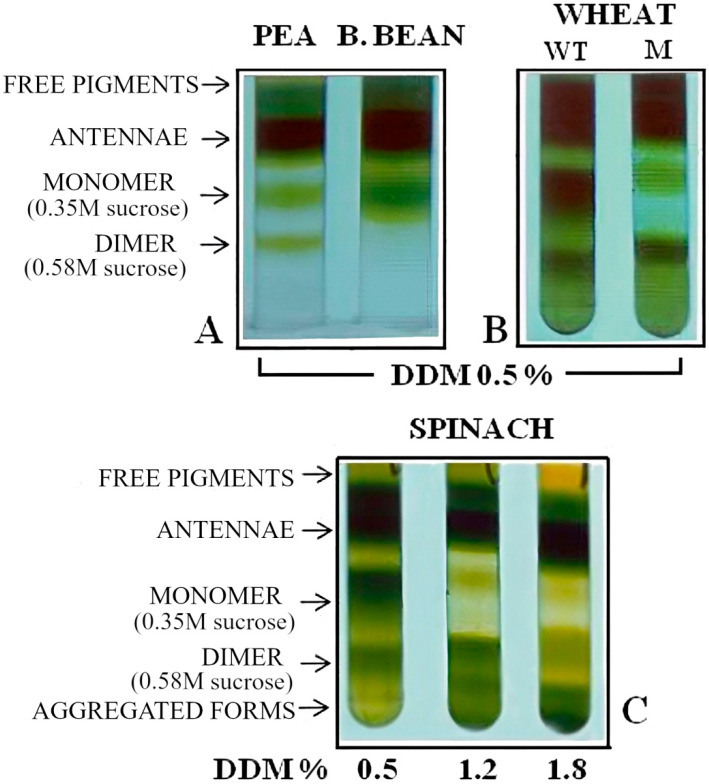
Photosystem II (PSII) particles were isolated from various species as indicated, and applied in sucrose gradient ultracentrifugation (SGU) as reported in the Material and Methods. For the gradients in A and B, the particles were solubilized at a concentration of 1 mg of Chlorophyll (Chl) in 0.3 mL 0.5% of n-dodecyl-β-d-maltoside (DDM). (**A**) Pea and broad bean PSII particles fractionation into free pigment–protein complexes, antennae, monomer at 0.35 M sucrose and dimer at 0.58 M sucrose. (**B**) Wheat and yellow-green wheat PSII particles fractionation into free pigment–protein complexes, antennae, monomer at 0.35 M sucrose and dimer at 0.58 M sucrose. (**C**) Spinach PSII particles fractionation into free pigment–protein complexes, antennae, monomer at 0.35 M sucrose and oligomeric aggregated cores. For the gradient in (**C**), 1 mg Chl of spinach particles were solubilized in 1 mL of increasing detergent concentration: 0.5%, 1.2%, 1.8% DDM. The experiments were repeated at least four times for each species, and a typical figure is presented.

Two large bands in the sucrose gradient were visible for *Pisum sativum*, *Spinacea oleracea*, *Vicia faba* and *Triticum durum*, which represent monomeric and dimeric PSII core populations centred at 0.35 and 0.58 M sucrose (Figure 1). HPLC size-exclusion analysis revealed their molecular masses as 250 ± 22 kDa and 470 ± 49 kDa, respectively, in agreement to that previously reported [29]. Similar results were obtained with *Medicago sativa*, *Phaseulus vulgaris* and *Zea mais* (data not shown). According to Boekema et al. [29], sodium dodecyl sulphate polyacrylamide gel electrophoresis (SDS-PAGE) and immunoblot analysis revealed that the two cores are composed of CP47, CP43, D2, D1, and Cyt b559 proteins, while the monomeric form occasionally includes the minor antenna CP29. However, PSI was not detected (data not shown).

We also found that the presence of the monomeric and dimeric PSII cores was not ubiquitous to all plant species. For instance, SGU of a yellow-green strain of *Triticum durum* revealed a preponderance of the dimeric PSII core (Figure 1B). P700 and Cyt f were found to be relatively absent in yellow-green wheat when the PSII particles of this low-chlorophyll wheat strain were compared with those of green wheat. The PSII electron transfer rate to ferricyanide was higher in the yellow-green strain compared to the green-type based on chlorophyll level, but similar to the two strain types when calculated based on leaf area (Table 1), in accordance with previous results [30]. However, PSII particles isolated from *Vicia faba* had only one PSII core band, which corresponded with the monomeric population as observed by HPLC-size exclusion (Figure 1A; see also Materials and Methods). These results from the yellow-green *Triticum durum* and the *Vicia faba* plants show that PSII can exist in vitro as a monomer or dimer. Depending on the solubilization settings chosen, two to four PSII core populations were evident, as shown in *Pisum sativum* (Figure 1C).

**Figure 2:**
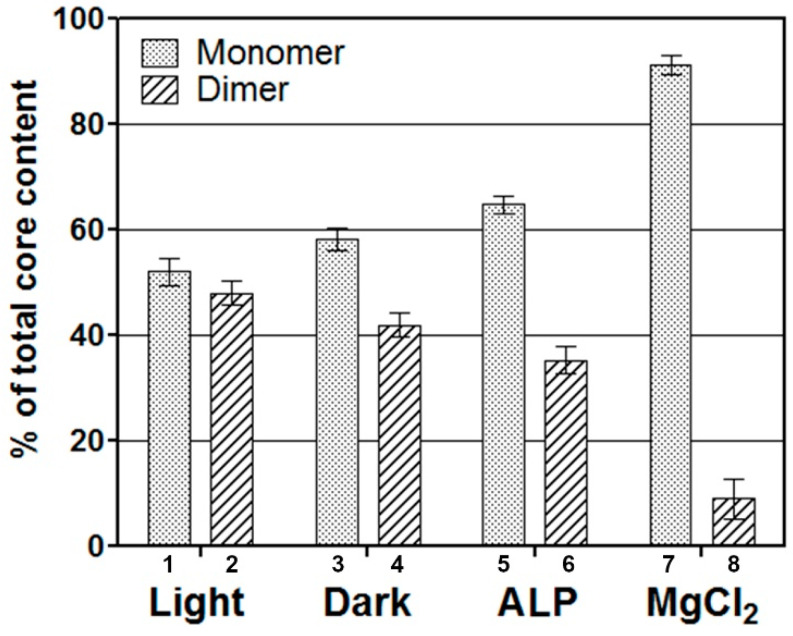
The photosystem II core population distribution in a sucrose gradient of solubilized pea PSII particles. PSII particles were isolated from ambient light and 8 h dark-adapted plants in the presence of a very faint green light (columns 1–2 and 3–4). Columns 5 and 6, particles treated with alkaline phosphatase (ALP). Columns 7 and 8, PSII particles isolated with a buffer containing MgCl_2_ (0.5 mM). The core distribution was measured as the percentage of chlorophyll (Chl) relative to the total chlorophyll in the cores. The values for Chl distribution are an average of three determinations. Six independent experiments were performed.

Next, we used detergent conditions that allowed the separation of the two PSII cores present in SGU at 0.35 and 0.58 M in seedlings of *Pisum sativum* treated as follows. Figure 2 shows the distribution of the two PSII core populations isolated in vitro in response to various treatments. PSII particles were isolated from *Pisum sativum* and left for 8 h at 100 μmol photons m^−2^ s^−1^ or for 8 h in the dark (Figure 2, columns 1–2 and 3–4). These data indicate that the distribution of the cores is regulated in part by the light conditions prior to extraction. Interestingly, when light-adapted PSII membranes were treated with an alkaline phosphatase (ALP) known to dephosphorylate the proteins, the distribution of the populations changed and the content of the least aggregated form greatly increased (Figure 2, columns 5,6).

It is known that thylakoid unstacking is induced under reduced magnesium content [9]. Therefore, *Pisum sativum* PSII particles were isolated also in a buffer containing MgCl_2_ at a concentration of 0.5 mM (Figure 2, columns 7–8). These particles showed a different core distribution compared to the PSII particles extracted at higher MgCl_2_, with mainly a large dimeric PSII core population having a mass of 270 ± 25 kDa (Figure 2, columns 7–8). 

Thus, the relative distribution of the aggregated core populations in vitro was found to be influenced by light and membrane stacking state prior to SGU. 

### 2.2. Distribution of PSII Core Populations Isolated from Stressed Pisum sativum 

**Figure 3:**
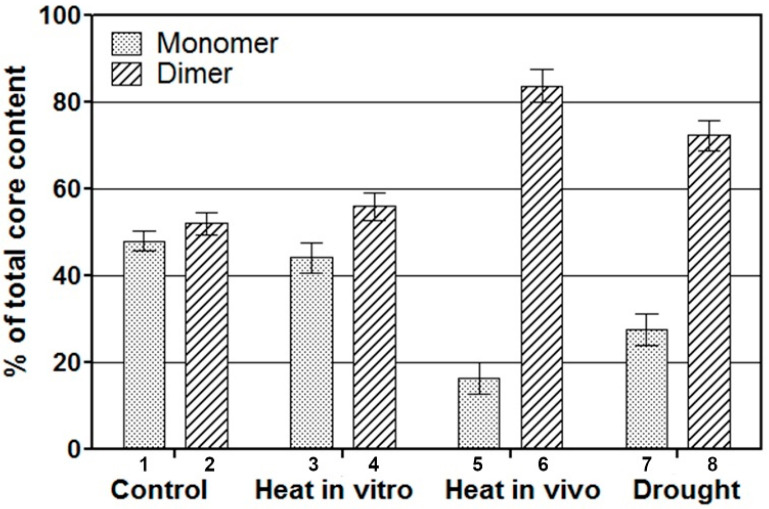
The response of the pea photosystem II (PSII) core population distribution to heat and drought stress analysed by sucrose gradient ultracentrifugation. The PSII particles were isolated from control plants and then subjected to 39 °C for 8 h (columns 3–4). PSII particles were isolated from pea plants grown at 25 ± 2 °C to 8 cm height and subjected as intact seedlings to 39 ± 2 °C for 8 h (columns 5–6). PSII particles isolated from drought-grown pea plants (columns 7–8). The experiment was triplicated.

To determine whether the oligomerization state of PSII depends on the conditions to which a plant is exposed, we analysed the distribution of the core populations in PSII particles isolated from stressed *Pisum sativum* seedlings. Typically, *Pisum* seedlings respond to stress conditions. Only small changes in SGU were observed in PSII-enriched membranes kept at 39 °C for 8 h after isolation (Figure 3). However, *Pisum* seedlings that were heat treated in vivo for 8 h prior to extraction of PSII-enriched particles had a different core distribution, with the dimeric form increasing from about 47% to 87% (Figure 3). Although visually the plants appear to be stressed, such conditions have been found not to deplete the protein content per leaf area [31]. The heat stress in vivo mildly altered PSII activity and the electron transport of PSII particles, isolated from stressed *Pisum* plants and measured from diphenylcarbazide (DPC) to the acceptor 2,6-dichlorophenolindophenol (DCPIP), remained high (97% of the initial value), while the F_v_/F_m_ fluorescence ratio was 0.853 ± 0.009 compared to 0.873 ± 0.003 in the control (Table 2). *Pisum* seedlings grown under drought conditions at a relative water content (RWC) of 70% maintained fluorescence activity, even when the PSII core content was partially reduced (Table 2). In such conditions, an increased content of the aggregated dimeric population was observed in SGU (Figure 3). Thus, the distribution of the two PSII core populations in vitro is altered in response to stress in vivo and leads to increased content of the aggregated forms.

### 2.3. Radiation Target Analyses of Thylakoids and PSII Preparations

RTA was used to determine the minimum PSII molecular size in *Pisum sativum*, *Spinacea oleracea*, *Vicia faba*, *Triticum durum* and the yellow-green *Triticum durum* that had different patterns of PSII core populations, as seen in sucrose gradient analysis (SGA) (Figure 1). According to radiation target theory, the enzyme activity is completely lost whenever a protein is hit due to a very high energy transferred through the chain. Therefore, we tested the effects of radiation using the F_v_/F_m_ fluorescence activity in the thylakoids, and the electron transfer activity from H_2_O or DPC to DCPIP in isolated enriched PSII particles. The minimum molecular mass required for these activities was calculated using the empirical relationship that considers the temperature dependence of the radiation inactivation Log MW = 5.89–log D37–0.0028T [32]. D37 is the dose of radiation in MRad required to inhibit the activity to 37% of the control, and T is the temperature in °C of the sample during irradiation.

**Figure 4:**
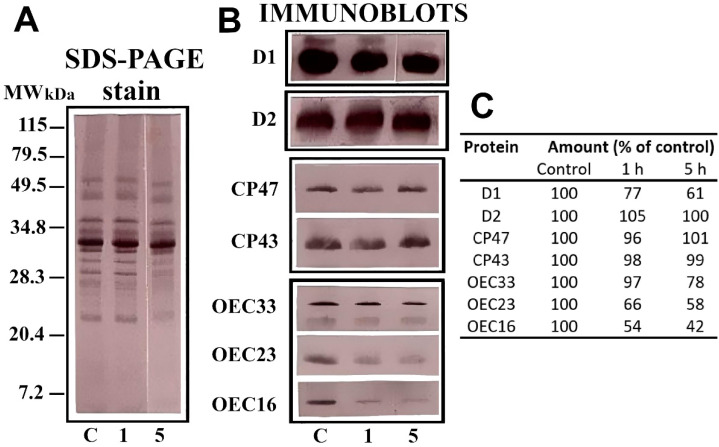
(**A**) Sodium dodecyl sulphate polyacrylamide gel electrophoresis (SDS-PAGE) analyses of pea photosystem II (PSII) particles: control and gamma-irradiated with a ^60^Co source of 0.803 Mrad h^−1^, according to the Materials and Methods. (**B**) Immunoblot analyses with polyclonal antibodies against the pea PSII proteins. (**C**) Densitometry-based relative amounts in immunoblots of control and irradiated PSII proteins. The values, in arbitrary units, are given as the percentage of the control obtained by dividing each individual polypeptide area of the immunoblot densitogram by the control area of polypeptide obtained from the corresponding densitogram at time zero. Experiments were repeated twice, and reproducible results were obtained. Mean values are given for three measurements of one typical preparation, standard error under 12%.

The thylakoids and PSII preparations were exposed to γ-radiation, and samples were taken at the indicated time intervals of 20–60 min. To determine the effects of radiation on PSII, we analysed the irradiated pea enriched PSII particles using polyclonal antibodies against the major PSII proteins. It was found that irradiation initially released the OEC proteins, and thereafter slightly affected the content of the D1 and D2 proteins, while the contents of the CP43 and CP47 proteins remained constant (shown in Figure 4).

**Figure 5:**
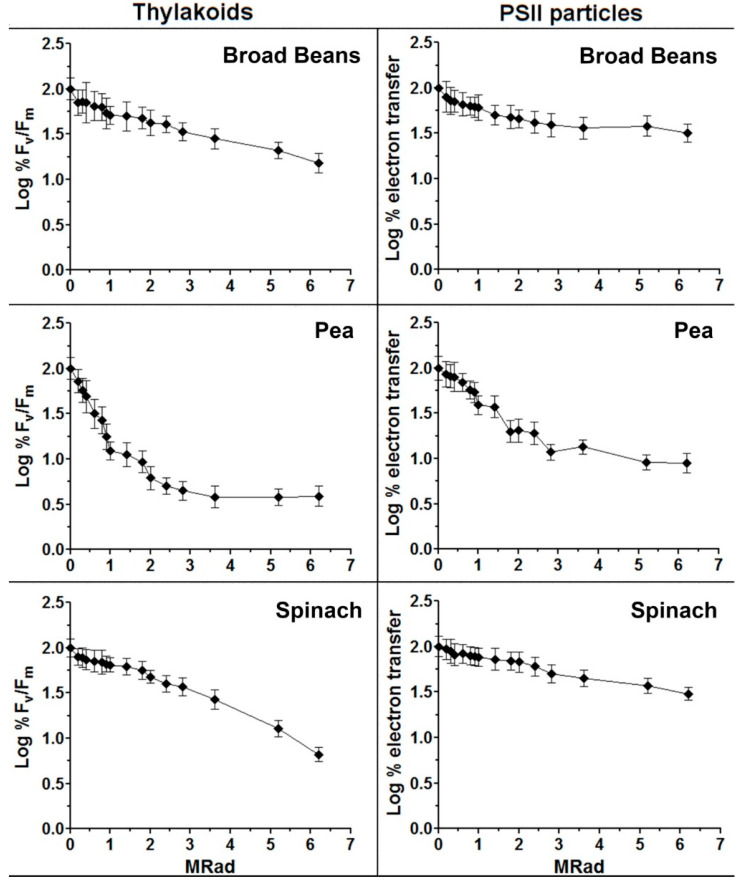
Radiation target analyses of thylakoids and photosystem II (PSII) particles isolated from broad bean, pea and spinach plant species. Initial PSII activity of the controls before irradiation averaged 0.856 ± 0.005 for fluorescence F_v_/F_m_ ratio, and 300 ± 28 μmol 2,6-dichlorophenolindophenol reduced per mg of chlorophyll h^−1^ for electron transfer activities. Each point is the mean of three measurements. The experiments were repeated at least five times using independent preparations for each species.

The loss of activity (reported as the Logarithm (Log) of the activity versus the dose of radiation) resulted in biphasic curves for the *Pisum* membranes (see Figure 5), possibly indicating a heterogeneous functional molecular mass. Relative to the other plants, *Vicia faba* and *Spinacea oleracea*, the curves were quite linear (Figure 5). This excluded a possible correlation between the biphasic nature of the pea RTA curves and the size heterogeneity detected by the sucrose gradient. PSII functional sizes calculated in the different plant membrane preparations are shown in Table 3. The RTA curves for pea were divided into two phases, leading to two different molecular weights (MW1 and MW2) (Figure 5 and Table 3).

The electron transfer activity in the presence of DPC, which is independent of the OEC activity, was measured in PSII membranes using a spectrophotometer. The use of the electron donor DPC coupled with DCPIP reduction allows the detection of PSII activity in sub-chloroplast fragments that have lost the ability to evolve oxygen. Thus, measuring electron transfer by DPC in comparison to that from water to DCPIP (Table 3) caused a reduction in the PSII functional size to about 70 kDa, corresponding to the mass of 33, 23 and 16 kDa proteins that make up the OEC. This observation suggested that the RTA method measures the whole PSII complex rather than only the D1 and D2 reaction centre II subunits known to be directly involved in the Q_B_-dependent electron transfer. 

The two core populations at 0.35 and 0.58 M sucrose were isolated from *Pisum* by SGU, and analysed by RTA. The molecular sizes of 260 ± 27 and 483 ± 34 kDa were found for the lower and higher density bands, respectively (Table 3). These values were similar to those determined by HPLC size exclusion, and thus validated the RTA methodology. 

### 2.4. Radiation Target Analyses of Intact Plant Leaves under Normal and Stress Conditions 

We also measured the PSII minimum functional size by the fluorescence in vivo specific to PSII activity in leaves of *Pisum sativum*, *Spinacea oleracea*, *Vicia faba*, *Triticum durum* and the yellow-green *Triticum durum.* Intact leaves were maintained at 0 ± 1 °C on ice during irradiation. Attention was paid to keep the leaves in the dark during all experimental phases (irradiation and fluorescence measurements) to avoid photoinhibition by the synergistic effect of low temperature and light. In fact, in control experiments, the F_v_/F_m_ ratio was not altered in leaves kept in the dark under the same conditions. All the tested plants had linear curves with a molecular size range of 75 ÷ 101 ± 18 kDa (Table 4).

Stressed *Pisum* seedlings were also analysed for minimum functional size by RTA (Table 4). The minimal functional PSII size doubled under heat stress and increased significantly under drought stress. Moreover, after recovery from stress, the minimum PSII functional size decreased again to the value close to the monomer (Table 4).

## 3. Discussion

Crystallographic analysis of the isolated core preparations has established the dimeric nature of PSII as a homodimer [19,33]. The present study addresses the question about the functionality of PSII under normal and stress conditions, both in vitro and in vivo. To address this, we analysed mainly *Pisum sativum*, but also several other plant species to highlight possible different behaviours.

Both monomeric and oligomeric forms of PSII were present in the isolated membrane fractions as previously reported [34]. Monomeric and dimeric PSII complexes isolated from *Spinacea oleracea* leaves by SGU were previously reported to have molecular masses of 450 ± 50 kDa and 250 ± 25 kDa [29]. We show here that similar values were obtained by RTA (Table 3). In the isolated complexes, the RTA analysis represents the size of the entire PSII complex, and the values are higher than those expected for the D1 and D2 reaction centre II proteins alone [26,27,28,35,36]. It was clear that the higher size measured by RTA was apparent in vitro rather than in vivo. This finding suggests that the entire molecule is so compact in vitro that a significant energy transfer is present among the various coupled units, rising to a higher molecular functional size.

It is known that the PSII structure is maintained by some lipids [37], and that the detergent can substitute for the membrane lipids [38]. The dimeric core has been shown to be surrounded by a monomolecular belt of detergent molecules under appropriate solubilising conditions [33]. The structural and functional role of phosphatidylglycerol in PSII and in PSII dimer–monomer interconversion is known [39,40,41,42,43]. Our finding of a main monomer core in vitro in *Vicia faba* with a low content of phosphatidylglycerol as compared to that in peas [44] is suggestive of different requirements for phospholipids. The fact that lipid composition changes under stress conditions has also been documented previously [45,46]. Our data support the importance of phospholipids in maintaining the structural integrity of the PSII complex and its oligomeric organization. Thus, the relative ratio of detergent to chlorophyll seems to be one factor that may strongly regulate PSII core aggregation in vitro. This observation has led other authors to conclude that the formation of several PSII cores could be an artefact, and that the monomeric form derives from the dissociation of the dimeric form. However, it is also noted that the occurrence of two PSII core populations in solubilized PSII particles has been previously attributed to the existence of both a monomeric and dimeric PSII in vivo [21]. Our analysis here has indicated that many plant species, namely, peas, spinach, broad beans, and wheat, contain both monomeric and dimeric PSII core populations in SGU when the n-dodecyl-β-d-maltoside (DDM) detergent concentration is below 1%. We also observed that monomeric and dimeric core populations are not ubiquitous in higher plants. For instance, both broad beans and a yellow-green wheat that maintain a high photosynthetic capacity in vivo were found to possess only one PSII core population in SGU, either a monomeric or a dimeric form.

Based on chlorophyll amount, the PSII electron transfer rate was higher in the yellow-green wheat strain, but when calculated based on leaf area, it was the same in both strain types (Table 1). Therefore, it has been previously suggested that the PSII, Cyt f and chlorophyll are not limiting factors of electron transport in wheat and, rather, the electron transport capacity may be more than that needed for driving photosynthesis [30,47].

Thus, it appears that both the monomeric and dimeric forms are sufficient in maintaining electron transport activity.

In addition, our data demonstrate that the relative distribution of the aggregated core populations responds to various treatments in vivo, and that the monomeric and dimeric forms of PSII do exist in vivo.

Interestingly, when PSII membranes were treated with an alkaline phosphatase (ALP) known to induce dephosphorylation of the protein thylakoids and modify their aggregation state [41,48,49], the distribution of the populations changed, and the content of the least aggregated form increased (Figure 2, columns 5–6).

Several biologically active systems, such as, for instance, invertase, adenylate cyclase and binding of thyroid stimulating hormone to receptors, have been previously analyzed with the radiation inactivation technique [24]. The great majority of target sizes were easily interpreted, while some target sizes were inconsistent with accepted values; however, many of these have been subsequently shown to be correct by independent measures [24].

Furthermore, also shown here is that the minimum functional size is flexible since mild stress conditions increase the content of aggregated PSII cores, leading to a doubled PSII minimum functional size, both in vitro and in vivo. Thus, the physiological state of a plant seems directly correlated to the distribution and formation of oligomeric PSII cores, as was indicated by heat- or drought-stressed peas. Moreover, PSII functions in vivo as a monomer, while under mild stress conditions it can function as a dimer.

**Figure 6:**
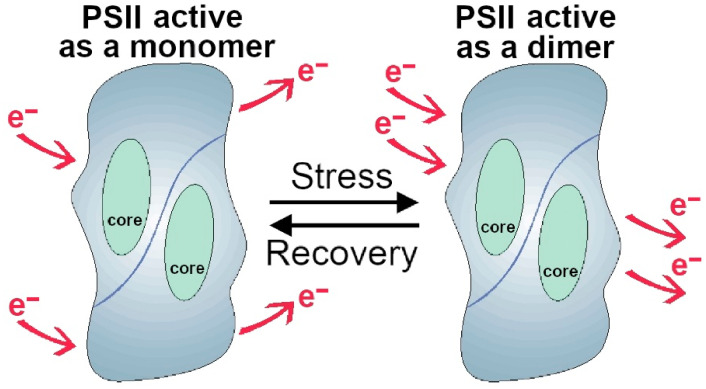
Hypothetical model of photosystem II (PSII) functional size interconversion from a monomer under normal conditions to a dimer under stress conditions.

Figure 6 presents a hypothetical model of PSII structure–function as regards the possible role of the dimeric structure. Under unfavourable and stress conditions, a tighter aggregation would be mediated by the modification of the relative content of lipids observed under stress; the process should help to keep Q_A_ and electron transfer components more closely associated, bypassing eventual damage points in the single unit. A comparison of the functional properties of the monomeric and dimeric reaction center complex is indicative of Q_A_ being more tightly bound in the dimeric form than in the monomeric one. Concerning the mechanism of electron transfer promoted by proteins, there are two possibilities: one in which the electron moves from bond to bond, and the other where the electron jumps into space by a tunnelling effect [7]. In the PSII dimeric structure, a vast space between the two joint cores has been observed [6]. Thus, we speculate that normally the electron moves from bond to bond in the PSII monomer, while in the dimeric form, under stress conditions, the electron can jump into space with a tunnelling effect (Figure 6). Future complex physical chemical analyses are necessary to prove or disprove this speculative model.

## 4. Materials and Methods

### 4.1. Plants and PSII Membrane Isolation 

Plants (Sementi Dotto, Italy): *Medicago sativa*, *Pisum sativum*, *Spinacea oleracea*, *Phaseulus vulgaris*, *Zea Mais*, *Vicia faba*, *Triticum durum* and a yellow-green strain of *Triticum durum* [30] were grown in soil in a greenhouse and harvested at the seedlings stage of 15 cm height and of 10 cm leaf length in the case of spinach. For dark-adapted membranes, after adaptation of plants to the dark for 8 h, the extraction was performed in the dark in the presence of a faint green light. For light-adapted plants at room light of about 50 μmol photons m^−2^ s^−1^, the buffers were at times supplied with 5 mM NaF to avoid dephosphorylation by phosphatase (s) during extraction in the dark. Thylakoids and PSII enriched particles were prepared at a temperature of 4 °C [50]. A chlorophyll-to-Triton X-100 ratio of 1:20 was used in the isolation of the PSII particles. 

The preparation of PSII monomeric and dimeric core complexes was as previously described [29], adapted with small modifications for the extraction of seedlings [51]. Isolated PSII particles were solubilized with DDM using the same chlorophyll to detergent ratio of 2:3 (1 mg Chl in 0.3 mL 0.5% DDM). Two PSII cores, consisting of monomeric and dimeric particles, were isolated from spinach by SGU using a 0–1 M linear gradient in sucrose dissolved in pH 6.3, 50 mM MES, 15 mM NaCl, 5 mM MgCl_2_ and 0.1% DDM, for 18 h at 5 °C and 39,000 rpm with a SW1 Beckman rotor. PSII particles isolated from treated plant species yielded PSII core bands. 

The core bands were characterized by SDS-PAGE. Immunoblots of the main PSII proteins (see sections below) and HPLC was performed on a Perkin Elmer apparatus, while size exclusion chromatography was done with a Beckman column TSK 4010 SW with a Pharmacia filtration kit as in previous studies [29].

For heat stress, pea seedlings were grown in a small green house at 25 °C and kept at 39 °C for 8 h in the dark at a controlled humidity of 50% [31]. For drought stress, pea seedlings in a greenhouse cabinet were deprived of water and analysed at a leaf relative water content of 70% [52].

### 4.2. Radiation Inactivation Analyses

RTA were performed as previously described [24,25]. High-energy γ-radiation was provided by a ^60^Co-source that provided a dose rate at the sample position of 0.803 Mrad h^−1^. Samples to be irradiated were placed in safe-cap Eppendorf vials in a Plexiglas box that contained a rack to keep a constant distance from the radiation source. The temperature was maintained at –18 ± 1 °C using a mixture of ice and sodium chloride which was replaced every 20 min. For intact leaves, the temperature was maintained at 0 ± 1 °C with ice. Attention was paid to keep the leaves in the dark during all experimental phases to avoid the induction of photoinhibition at the low temperature [53]. 

In control experiments, there was no change in the maximum quantum yield of photochemistry of PSII (F_v_/F_m_) in leaves maintained at the same low temperature and under the same conditions. 

There was no detectable decrease in the Chl concentration or bleaching of the samples due to γ-irradiation. 

### 4.3. Photosystem II Activity, Chlorophylls, P700 and Cyt b559 Content 

Fluorescence activity was measured as the maximum quantum yield of photochemistry of PSII (F_v_/F_m_) using a PEA apparatus (Hansatech Instruments Ltd., Pentney, UK) on irradiated samples kept at 5 °C in the dark. Chlorophyll content was calculated using the Lichtenthaler method [54]. For P700 and Cyt f measurements, leaves were homogenized in a cold mortar at pH 7.2 with 50 mM KH_2_PO_4_ buffer, filtered and centrifuged at 6000× *g* for 5 min [30]. The pellet was washed and resuspended in the same buffer. This volume was divided into two vials for different analyses. For P700 measurements, one part of the sample was diluted in 50 mM KH_2_PO_4_ (pH 7.3) containing 0.5 sucrose and 0.1% (*w*/*v*) Triton with a concentration of 40 µg Chl mL^−1^. For Cyt f measurement, the second fraction of suspended thylakoids was diluted in 50 mM KH_2_PO_4_, 2 mM EDTA (pH 6.8), 0.3 M mannitol, 1 mM MnCl_2_, 1 mM MgCl_2_ and 0.5% (*w*/*v*) Triton to give a final concentration of 60 µg Chl mL^−1^. After 30 min at 25 °C, the samples were centrifuged at 3000× *g* for 5 min. P700 and Cyt f (3 mL) were measured by the oxidized-minus-reduced difference spectra using a Shimadzu UV-120-02 spectrophotometer. P700 measurement was carried out to determine the differences between the ox-red spectrum involving the addition of 20 µL of 0.05 M potassium ferricyanide to one cuvette and 10 µL of 0.20 M of sodium ascorbate to the other. The difference spectrum was determined between 750–600 nm, and an extinction coefficient of 64 cm^−1^ mM^−l^ was used for calculations. The oxidized-minus-reduced difference spectrum of Cyt f was obtained between 580 and 520 nm by adding 10 µL of 0.08 M potassium ferricyanide to one cuvette and 10 µL of 0.3 M sodium ascorbate to the other. An extinction coefficient of 17.7 cm^−1^ mM^−l^ was used for the calculations.

The electron transfer activity was measured in PSII particles in the presence of 100 μM of DCPIP with or without 150 μM of DPC using a spectrophotometer at 600 nm [30]. PSII cores were monitored by HPLC size exclusion chromatography (Zorbax GF450 column) using 200 mM Tris, 1 mM DDM mobile phase [29,39].

### 4.4. SDS-PAGE and Immunoblot Analyses

The protein composition of isolated membranes and PSII particles was analysed by SDS–PAGE and gels were stained with Coomassie Blue.

Immunoblotting was carried out after the transfer of proteins separated by denaturing 12–17% polyacrylamide gels to nitrocellulose filters [52]. The immuno-complexes were detected using anti-rabbit secondary antibodies, coupled to alkaline phosphatase [55]. Band intensity was measured densitometrically using a Shimadzu CS 930 densitometer (Shimadzu, Tokyo, Japan).

Polyclonal antibodies against the OEC extrinsic proteins (33, 23, and 16 kDa) and against the core proteins, namely, D1, D2, CP43, and CP47, were kindly supplied by Prof. Udo Johanningmeier (Institute of Plant Physiology, Martin-Luther University Halle-Wittenberg, Halle-Saale, Germany). 

All experiments were repeated at least three times, and one typical example is shown in Figure 4.

### 4.5. Statistical Analysis

All statistical tests were performed using analysis of variance (ANOVA). The statistical significance of differences was evaluated by *p*-level at a significance level of *p* < 0.05, after the homogeneity tests.

## 5. Conclusions

We used RTA to determine the functional size of PSII in vitro and in vivo. We show that in comparison to the SGU of PSII cores in vitro, PSII in vivo also functions as a monomer under normal conditions. Moreover, under heat and drought stress conditions, the dimeric PSII functional structure is predominant in vivo, while the monomer form increases again after recovery from stress.

We suggest that under stress conditions, a tighter aggregation is likely mediated by the modification of some lipids, which helps to keep Q_A_ and electron transfer components more closely associated. Since the minimal PSII function seems to be flexible depending on the conditions employed, the formation of the dimer under stress, by passing eventual damage points in the single unit, perhaps could be a possible mechanism of resistance to stress conditions.

## Figures and Tables

**Table 1:** Photosynthetic characteristics of wheat and yellow-green wheat as a ratio between the wheat/yellow-green wheat are shown.

	Ratio Wheat/Yellow-Green Wheat
Total chlorophyll	2.52 ± 0.40
Chlorophyll a/b	1.31 ± 0.12
P700	2.00 ± 0.31
Cyt f	1.40 ± 0.09
Electron transport	
on chlorophyll basis	0.39 ± 0.06
on leaf basis	0.98 ± 0.05

**Table 2:** Photosystem II activity in peas under stress (heat and drought) measured as the fluorescence F_v_/F_m_ ratio in leaves and as electron transfer in isolated PSII particles. RWC, relative water content.

	F_v_/F_m_ Pea Leaves	ET Percentage of Control (from DPC to DCPIP) Pea PSII Particles
**Control**	0.873 ± 0.003	100 ± 3
**Heat stress** 39 °C	0.853 ± 0.009	97 ± 6
**Recovery** (2 days at 25 °C)	0.869 ± 0.004	99 ± 3
**Drought** RWC 70%	0.866 ± 0.015	99 ± 8
**Recovery** (3 days after watering)	0.861 ± 0.05	101 ± 8

**Table 3:** Radiation target analyses of thylakoids, photosystem II (PSII) particles and PSII cores. The fluorescence and electron transfer from H_2_O or diphenylcarbazide (DPC) to 2,6-dichlorophenolindophenol (DCPIP) were measured as reported in the Materials and Methods. Before irradiation, the initial PSII activity of the controls averaged 0.856 ± 0.005 for fluorescence F_v_/F_m_, and 300 ± 28 μmol DCPIP reduced per mg of Chl h^−1^ for electron transfer activities. The molecular sizes (MW) in kDa were calculated according to Beauregard and Potier [32]. The experiments were repeated at least five times in independent preparations for each species.

**Thylakoids (F_v_/F_m_)**		
**Plants**	**MW**	
Spinach	334 ± 39	
Broad bean	270 ± 31	
Wheat	253 ± 22	
Yellow-green wheat	Nd	
Peas-MW 1	1300 ± 63	
Peas-MW 2	291 ± 23	
**PSII particles (Electron Transfer)**		
**MW**		
	**Electron Transfer**	
**Plants**	**H_2_O → DCPIP**	**DPC → DCPIP**
Spinach	226 ± 26	177 ± 18
Broad bean	250 ± 22	186 ± 21
Wheat-wild-type	205 ± 16	171 ± 25
Wheat-mutant	232 ± 31	155 ± 15
Peas-MW 1	1200 ± 68	548 ± 44
Peas-MW 2	249 ± 27	172 ± 19
**PSII Cores (Electron Transfer DPC → DCPIP)**		
**Peas**	**MW**	
PSII Core monomer	260 ± 27	
PSII Core dimer	483 ± 34	

**Table 4:** Radiation target analyses (RTA) of intact seedlings leaves. In control experiments, there was no change in the F_v_/F_m_ fluorescence ratio in leaves kept at the same low temperature and conditions. Functional molecular sizes (kDa) of PSII were determined by the Beauregard and Potier relationship [32]. The experiments were repeated four times. R: linear correlation coefficient.

**Intact Leaves**	**PSII Size Measured by RTA**	**R^2^**
Broad beans	101 ± 18	0.94
Spinach	97 ± 18	0.96
Peas	75 ± 13	0.95
Peas after heat stress	160 ± 17	0.93
Peas after recovery from heat stress	80 ± 11	0.96
Peas after drought	129 ± 21	0.92
Peas after recovery from drought	122 ± 17	0.96

## Data Availability

The data that support the findings of this study are available from the corresponding author upon reasonable request.

## Sample Availability

Not available.
